# Glossitis in Typhoid Fever, with Report of a Case

**Published:** 1898-08

**Authors:** Thomas McCrae


### GLOSSITIS IN TYPHOID FEVER, WITH REPORT OF A CASE.

The cases of the concurrence of this complication in typhoid fever reported in the literature appear to be very few, the condition itself being a comparatively rare one. In over 700 cases of typhoid fever treated in the Johns Hopkins Hospital, this is the first time that this condition has been found. In the case to be reported it is of especial interest, in that it occurred during convalescence from the original attack and ushered in a relapse.

There are numerous references by the older writers to the association of glossitis with the eruptive fevers. Thus, Kerr, writing on glossitis in a Cyclopedia of Practical Medicine, published in London, in 1833, speaks of "tumefied states of the tongue which occur in typhoid and various fevers attended with an atonic condition of the system." There are numerous references to glossitis coming on during the course of or in convalescence from acute febrile diseases. Clark, in his work on the tongue, says, "Indeed, slight attacks of intercurrent glossitis are not infrequent in the course of eruptive fevers." But neither he nor Butlin, in his "Diseases of the Tongue," refers to any instances in which it occurred with typhoid fever. No reference to the association of the two was to be found in any of the text-books of medicine. Hoffman in his book on the pathological conditions in typhoid fever does not speak of it. Sorel, in his statistics of 871 cases, does not report its occurrence, nor Freundlich, in a statistical report of cases in Freiburg, Renou and Gallety-Bosviel, in special articles on the tongue and mouth in typhoid fever, do not mention glossitis. The reports of Berg, Jenner, and Studer, embracing the reports of the examinations of 1,984 cases, do not speak of it. Holscher, in the statistics of 2,000 cases, speaks of "purulent infiltration" of the tongue in three cases, while Dopfer in 927 cases found the same condition in two cases.

Nichols has reported a case of "septic infection in typhoid fever," in which two days before death swelling of the right half of the tongue was noted. The case came to autopsy, and the tongue was found red, swollen, and glazed in its right half. On section it showed hemorrhages and small abscesses. Cultures showed streptococci, staphyloccocci and the colon bacillus. This may, perhaps, be the same condition as Holscher and Dopfer have spoken of as "purulent infiltration" of the tongue.

The case reported is from Dr. Osler's clinic in the Johns Hopkins Hospital:

W. U., aged twenty-seven, white, dredger. Admitted, on November 27, 1897, with a mild attack of typhoid fever. The previous history was unimportant. The attack was quite characteristic; fever, rose spots, enlarged spleen, and the Widal reaction all being present. The temperature fell to normal on the sixteenth day and he made an uninterrupted recovery. He was discharged on December 31, 1897, on the thirty-seventh day of his disease, and after twenty-two days of normal temperature. He seemed perfectly well on discharge.

On January 3, 1898, the fourth day after leaving the hospital, he was readmitted, complaining of pain in the throat with soreness and swelling of the tongue. He gave a history of having felt well until January 2, when he had a chill, soon followed by pain in the head and throat. Swelling of the tongue and behind the jaw accompanied by pain on swallowing also came on There was no history of the taking of mercury or the application of any irritant. His condition rapidly grew worse until his admission.

On admission—temperature 104.2°, pulse 100, face flushed, the neck full and swollen at the angles of the jaws—the mouth presented a striking picture. The tongue was much swollen, protruding between the teeth and preventing the closing of the mouth. There was a profuse constant flow of saliva. The tongue was red, inflamed, symmetrically enlarged, markedly tender, and somewhat indurated as far back as could be felt. No spot of softening could be found. The throat could not be seen. Swallowing was difficult. Cultures were taken from the left half of the tongue, by Dr. Gwyn. Bleeding followed the punctures. On the following day the swelling was less, and the left half was rather smaller than the right, due, probably, to the bleeding following the punctures. Two days later there was less swelling, less pain, and the mouth could be closed. Three days later the tongue was practically normal.

The temperature, which on admission was 104.2°, fell to normal on the day after admission and then rose gradually each day until it reached 104° on January 7. With this he had a typica relapse, with continued fever, rose spots, and enlarged spleen. This lasted for about two weeks, and was mild throughout. The temperature fell to normal on the sixteenth day of the relapse, and he was discharged well on January 26. The cultures from the tongue were negative.

In this case after twenty-four days of normal temperature the glossitis seemed to be first symptom of the relapse. The relapse itself was mild save for the severe onset, and as soon as the swelling subsided the patient had no further trouble in swallowing or distress of any kind. The diminution of the swelling in the left half of the tongue after the blood removed in taking the cultures, supports the value of the treatment advised in severe cases: namely, free incisions into the substance of the tongue.—*Thomas McCrae, M. D., in Johns Hopkins Hospital Bulletin.*

